# A Low-Producing Haplotype of Interleukin-6 Disrupting CTCF Binding Is Protective against Severe COVID-19

**DOI:** 10.1128/mBio.01372-21

**Published:** 2021-10-12

**Authors:** Tao Chen, Yu-Xin Lin, Yan Zha, Ying Sun, Jinxiu Tian, Zhiying Yang, Shan-Wen Lin, Fuxun Yu, Zi-Sheng Chen, Bo-Hua Kuang, Jin-Ju Lei, Ying-jie Nie, Yonghao Xu, Dong-Bo Tian, Ying-Zi Li, Bin Yang, Qiang Xu, Li Yang, Nanshan Zhong, Meizhen Zheng, Yimin Li, Jincun Zhao, Xiang-Yan Zhang, Lin Feng

**Affiliations:** a State Key Laboratory of Oncology in South China, Collaborative Innovation Center for Cancer Medicine, Department of Experimental Research, Sun Yat-sen University Cancer Center, Guangzhou, China; b State Key Laboratory of Respiratory Disease, National Clinical Research Center for Respiratory Disease, Guangzhou Institute of Respiratory Health, the First Affiliated Hospital of Guangzhou Medical University, Guangzhou, China; c NHC Key Laboratory of Pulmonary Immunological Diseases, Guizhou Provincial People’s Hospital, Guizhou University, Guizhou, China; d Yangjiang Key Laboratory of Respiratory Disease, Department of Respiratory Medicine, People’s Hospital of Yangjiang, Yangjiang, Guangdong, China; e Department of Respiratory Medicine, The Sixth Affiliated Hospital of Guangzhou Medical University, Qingyuan People’s Hospital, Qingyuan, China; f Department of Biology, Southern University of Science and Technology, Shenzhen, China; Universidade de Sao Paulo

**Keywords:** COVID-19, CTCF, interleukin-6, genetic polymorphisms

## Abstract

Interleukin6 (IL-6) is a key driver of hyperinflammation in COVID-19, and its level strongly correlates with disease progression. To investigate whether variability in COVID-19 severity partially results from differential *IL-6* expression, functional single-nucleotide polymorphisms (SNPs) of *IL-6* were determined in Chinese COVID-19 patients with mild or severe illness. An Asian-common *IL-6* haplotype defined by promoter SNP rs1800796 and intronic SNPs rs1524107 and rs2066992 correlated with COVID-19 severity. Homozygote carriers of *C-T-T* variant haplotype were at lower risk of developing severe symptoms (odds ratio, 0.256; 95% confidence interval,  0.088 to 0.739; *P *= 0.007). This protective haplotype was associated with lower levels of *IL-6* and its antisense long noncoding RNA *IL-6-AS1* by *cis*-expression quantitative trait loci analysis. The differences in expression resulted from the disturbance of stimulus-dependent bidirectional transcription of the *IL-6*/*IL-6-AS1* locus by the polymorphisms. The protective rs2066992-*T* allele disrupted a conserved CTCF-binding locus at the enhancer elements of *IL-6-AS1*, which transcribed antisense to *IL-6* and induces *IL-6* expression in inflammatory responses. As a result, carriers of the protective allele had significantly reduced *IL-6-AS1* expression and attenuated *IL-6* induction in response to acute inflammatory stimuli and viral infection. Intriguingly, this low-producing variant that is endemic to present-day Asia was found in early humans who had inhabited mainland Asia since ∼40,000 years ago but not in other ancient humans, such as Neanderthals and Denisovans. The present study suggests that an individual's *IL-6* genotype underlies COVID-19 outcome and may be used to guide IL-6 blockade therapy in Asian patients.

## INTRODUCTION

Coronavirus disease 2019 (COVID-19) is an infectious respiratory disease caused by severe acute respiratory syndrome coronavirus 2 (SARS-CoV-2), an RNA virus spreading rapidly. It is the third most highly pathogenic coronavirus after SARS-CoV and Middle East respiratory syndrome (MERS)-CoV. The clinical presentation of COVID-19 varies between individuals. Most patients develop mild or no illness, but a minority of patients suffer from severe COVID-19, including acute respiratory distress syndrome (ARDS) and systemic inflammation. According to data from China, 84.3% with COVID-19 developed mild illness and 15.7% developed severe disease (1). COVID-19 also exhibited striking geographical and ethnic disparities. The lowest fatality rates were observed in East and Southeast Asia, while the fatality rates in some European and American countries were up to ∼100-fold higher than those in East Asia (according to data from the Johns Hopkins University Coronavirus Resource Center, https://coronavirus.jhu.edu/data/mortality). Variability in COVID-19 severity could be explained by many factors, including anti-epidemic measures and medical resources in different countries as well as the individual’s age, sex, and comorbidity (2). In addition, an individual’s genetic background may also affect COVID-19 vulnerability, especially genetic variations that explain different immune responses to coronaviruses, such as Toll-like receptors (TLRs) (3). Importantly, a genome-wide association study (GWAS) reported that a Neanderthal-derived region of chromosome 3 was the major genetic risk factor for severe COVID-19 in populations in Italy and Spain (4, 5). However, the frequency of the Neanderthal risk haplotype varies widely between different populations and is almost absent from East Asia, suggesting that the genetic contribution of this haplotype to COVID-19 severity is small in East Asians. Given the immune-related risk loci often show heterogeneity between populations, the genetic susceptibility of COVID-19 in East Asian populations is worthy of investigation.

Interleukin-6 (IL-6) is a multifunctional cytokine secreted from many cell types, including monocytes/macrophages, dendritic cells, fibroblasts, endothelial cells, and B and T cells, in response to inflammatory stimuli and viral infections. IL-6 is a key player in the exacerbated inflammatory response in SARS-CoV-2 infection (6). Elevated serum concentrations of IL-6 and other cytokines are hallmarks of severe COVID-19 (7). The excessive IL-6 creates high levels of inflammation that often result in the development of ARDS, which is the leading cause of death from COVID-19 and other coronavirus-related diseases, including SARS and MERS (8–11). Because IL-6 level strongly correlates with disease progression, the serum concentration of IL-6 has been used to monitor the severity of COVID-19 (7, 12, 13), and it acts as a predictor of mortality (14).

IL-6 expression is highly influenced by polymorphisms in promoter and regulatory regions. Some of these single-nucleotide polymorphisms (SNPs) vary significantly between different ethnic populations and are related to disparities in the response to a number of pathogens. For example, *IL-6* promoter SNP rs1800796, which is highly prevalent in Asia, has been proposed to explain ethnic-specific susceptibility to some infectious diseases. Individuals of Hmong ancestry harboring the *IL-6* variant haplotypes rs1800796, rs1524107, and rs2066992 had higher risk of serious fungal infection than individuals of European ancestry harboring the wild-type (WT) haplotype (15). For hepatitis B virus (HBV) infection that is endemic to Asian countries, the rs1800796 *C*/*C* variant genotype was found to be detrimental to spontaneous clearance of HBV in Chinese (16) and associated with increased risk of chronic HBV infection in Malaysians (17). *IL-6* promoter SNPs rs1800795 and rs1800797 are exclusively polymorphic in Caucasians. The *G*/*G* genotype of rs1800795 was protective against pneumococcal pneumonia in white Spanish patients (18, 19), but the *G*-*G* alleles at rs1800795 and rs1800797 favored a worse evolution of HCV chronic infection in Italians (20) and also conferred susceptibility to human papillomavirus (HPV)-associated cervical cancer in North Indians (21). In summary, the impact of *IL-6* polymorphisms in infectious diseases varies depending on the causative pathogens and ethnicities.

The present study determined the influence of *IL-6* polymorphisms on the severity of COVID-19 in Chinese patients. We found an Asian-common haplotype, *C*-*T*-*T*, represented by variant allele rs1800796, rs1524107, and rs2066992 loci, favored a better outcome of SARS-CoV-2 infection, associated with a reduced expression of *IL-6* by inflammatory stimuli and viral infection. Our results highlight the potential of these *IL-6* SNPs as biomarkers in the prognosis and treatment of COVID-19 in populations with Asian ancestry.

## RESULTS

### An Asian-common haplotype of *IL-6* correlates with lower *IL-6* expression.

**FIG 1:**
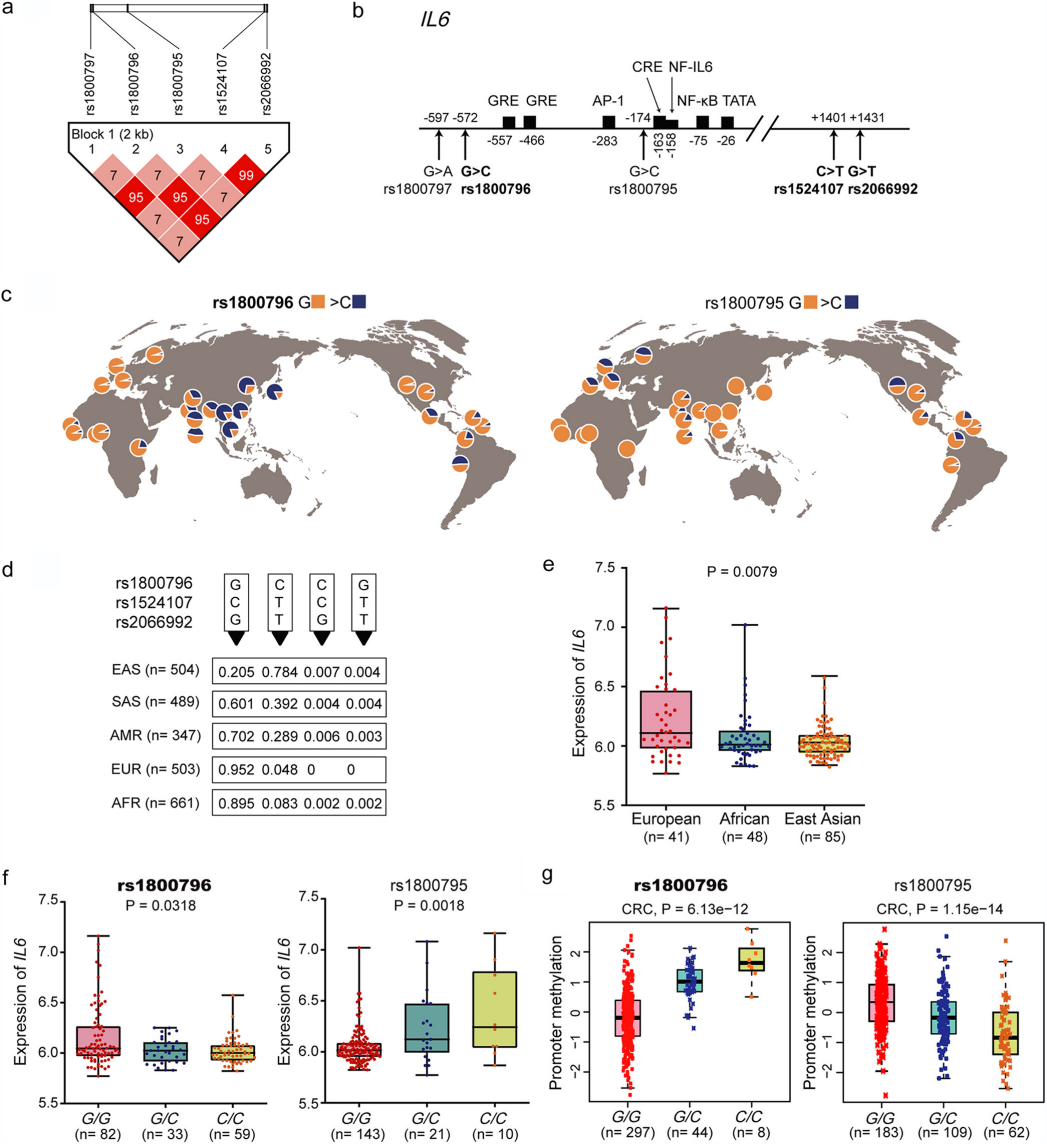
Linkage disequilibrium (LD) and geographical distribution of SNPs in the *IL-6* promoter that closely correlate with expression. (a) LD plot of promoter SNPs rs1800797, rs1800796, and rs1800795 and two SNPs in LD with rs1800796. The candidate SNPs are in boldface text. The LD values for each SNP based on the genotype information of 2,504 individuals from different worldwide populations (1000 Genomes Project). The blocks are shaded corresponding to the LD values, and the numbers in squares show the percent *r*^2^ between SNPs with incomplete LD. (b) Schematic representation of human *IL-6* gene. The polymorphic sites and transcription factor binding sites are indicated. Positions are relative to the transcription start site (TSS) of *IL-6*. GRE, glucocorticoid response element; CRE, the cAMP response element. (c) Geographic distribution of rs1800796 and rs1800795. Pie charts indicate allele frequency at each SNP. Frequency data were determined as for panel a and exported by GGV. (d) Haplotype of rs1800796, rs1524107, and rs20669992 in five superpopulations. G-C-G and C-T-T are the most common haplotypes in the populations. EAS, East Asian; SAS, South Asian; AMR, admixed American; EUR, European; AFR, African. (e) Normalized *IL-6* mRNA expression in lymphoblastoid cell lines (LCLs) derived from three major world populations of European, African, and East Asian (Chinese and Japanese) ancestry. Data are from the International HapMap Project (*N* = 174). Statistical analysis was performed by Kruskal-Wallis test. (f) Allelic expression data set for *IL-6* gene was generated from the HapMap panel of LCL cell lines as in panel e. Statistical analysis was performed by Kruskal-Wallis test. (g) Genotypes of rs1800796 (chr7:22766246, hg19) and rs1800795 (chr7:22766645, hg19) and the methylation levels at probe cg26061582 (chr7:22766209, hg19) in colorectal cancer (CRC). Data are from Pancan-meQTL.

**FIG 2:**
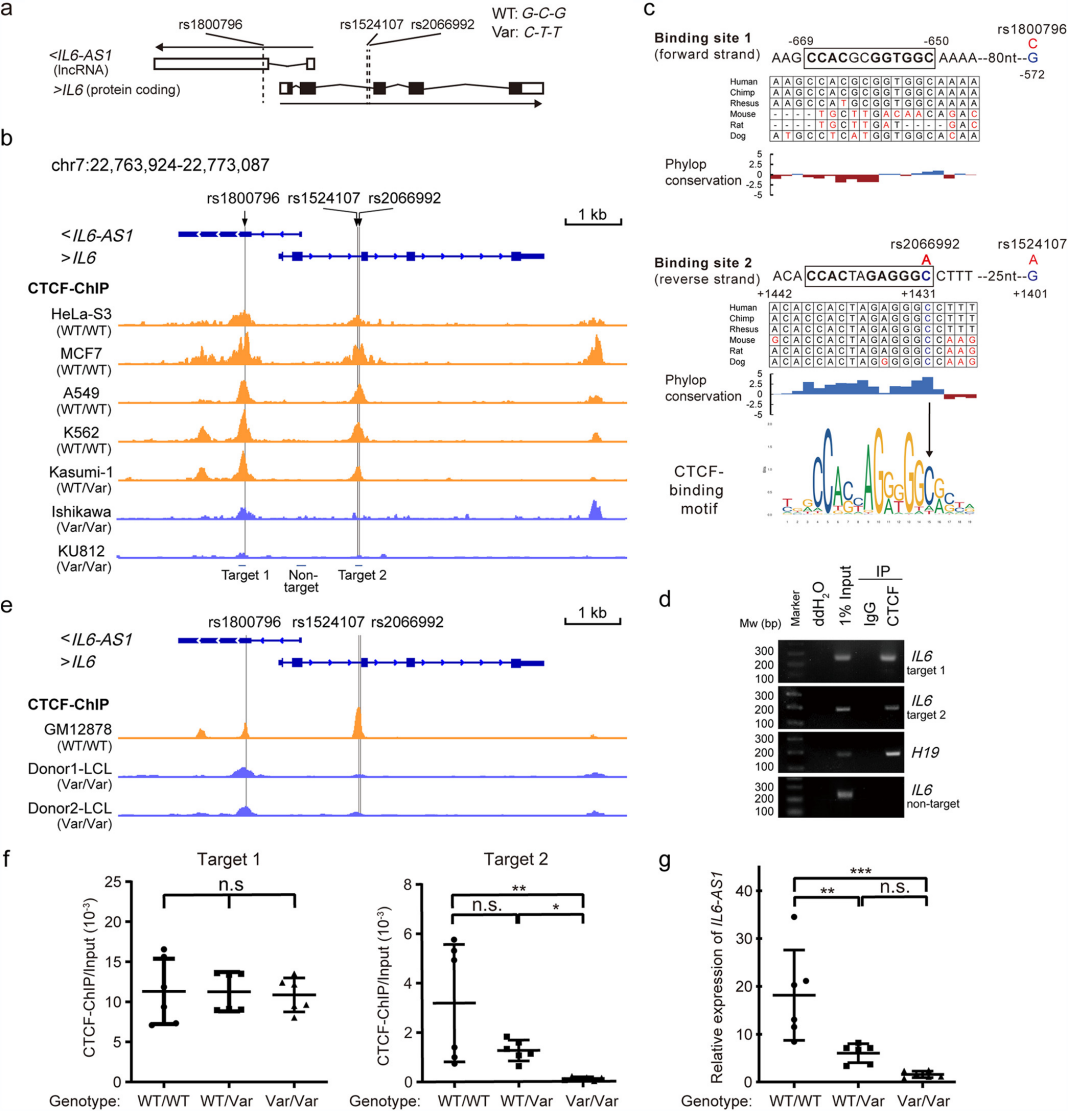
Haplotypes of rs1800796, rs1524107, and rs20669992 affect the enrichment of CTCF in the *IL-6*/*IL-6-AS1* locus. (a) Genomic locations of rs1800796, rs1524107, and rs20669992 in *IL-6* and its antisense lncRNA *IL-6-AS1*. Exons are marked with boxes, in which untranslated regions are shown as open boxes, and coding regions are marked with black boxes. (b) The susceptibility SNPs rs1800796, rs1524107, and rs2066992 reside in or close to the CTCF-binding regions of the *IL-6*/*IL-6-AS1* locus. The ChIP-PCR fragments used in panel d are marked with lines below the gene. (c) The location of CTCF-binding sites in the *IL-6* gene. Human CTCF binding motif logo was from JASPAR^2020^, and the 12-bp consensus sequences of CTCF-binding sites are in boxes. The wild-type allele is in blue and variant allele in red. Phylop basewise conservation score derived from 100 vertebrate species is shown. (d) ChIP-PCR in PBMCs of a donor with wild-type genotype at the candidate SNPs using anti-CTCF antibody or control IgG. *H19* was used as a positive control. MW, molecular weight; ddH_2_O, double-distilled water. (e) CTCF binding intensity at *IL-6/IL-6-AS1* locus in LCL cell lines with different genotypes at the loci of interest. (f) ChIP-qPCR assay of CTCF binding at two target regions of *IL-6* locus in PBMCs of healthy donors with different genotypes. Each assay was performed for each donor (WT/WT, *n* = 2; WT/Var, *n* = 2; Var/Var, *n* = 2) in technical triplicate. Data represent means ± SD per group. One-way ANOVA with Bonferroni correction for multiple comparisons. (g) Basal level of *IL-6-AS1* in PBMCs of donors as in panel f. Each assay was performed for each donor in technical triplicate. Relative expression of *IL-6-AS1* is normalized to GAPDH. Data represent means ± SD per group. One-way ANOVA with Bonferroni correction for multiple comparisons was used. *, *P *< 0.05; **, *P *< 0.01; ***, *P *< 0.001; n.s., not statistically significant.

*IL-6* expression is highly influenced by polymorphisms in the promoter region. Numerous studies revealed that three SNPs, rs1800797 (−597 *G *>* A*), rs1800796 (−572 *G > C*), and rs1800795 (−174 *G > C*), in the proximal promoter, affect the transcription and secretion of IL-6 and were associated with susceptibility to various infectious agents, including bacteria, fungi, and viruses (15, 17, 18, 21, 22). We explored the SNPs and haplotype diversity in different populations worldwide provided by the 1000 Genomes Project (23). Haplotype analysis based on the genotype information of 2,504 individuals established that the three promoter SNPs exhibited distinct linkage disequilibrium (LD) patterns. Rs1800795 and rs1800797 were in high LD (*r*^2^ > 0.95), and rs1800796 was strongly linked with rs1524107 and rs2066992 (*r*^2^ > 0.95; Fig. 1a), with the latter two SNPs located in the intron of *IL-6* (Fig. 1b and 2a). Notably, the three promoter SNPs exhibited striking ethnic disparities in comparisons of population allele frequencies worldwide. rs1800796 variation was highest in East Asians (79%), followed by South Asians (39%) and Americans (30%), but was rare in Africans (10%) and Europeans (5%) (Fig. 1c, left). In contrast, rs1800797 and rs1800795 were highly polymorphic in Caucasians but almost monomorphic in Asians and Africans (Fig. 1c, right). Because of the high LD of rs1800796, rs1524107, and rs2066992, two typical haplotypes exist worldwide. The wild-type haplotype *G-C-G* was common in Europeans and Africans, and the derived haplotype *C-T-T* was most prevalent in East Asians (Fig. 1d).

Having shown the ethnic disparities in *IL-6* functional SNPs, we then compared *IL-6* expression levels in the three major world populations, European, African, and East Asian, by utilizing microarray expression data from the HapMap LCLs (lymphoblastoid cell lines, the EBV-transformed human B-cell lines), an international project providing genotypic data on individuals of major world populations (24). A significant higher expression level of *IL-6* was observed in European population, followed by Africans, and East Asians had the lowest *IL-6* levels (Fig. 1e). We then attempted to establish the associations between the genotypes of Asian-common haplotype (rs1800796 as a tag SNP) and Caucasian-common haplotype (rs1800795 as a tag SNP) with *IL-6* levels. Expression levels of *IL-6* in LCLs from the HapMap Project with genotypic data at the candidate loci were analyzed. The results suggested that rs1800796 variant allele *C* associated with lower *IL-6* expression, while rs1800795 variant allele *C* correlated with higher *IL-6* expression than their ancestral allele, *G* (Fig. 1f). Furthermore, higher DNA methylation in the *IL-6* promoter was found in a minor allele (*C*) at rs1800796; in contrast, the derived *C* allele at rs1800795 was associated with lower DNA methylation in colorectal cancer tissues (Fig. 1g) and another cancer type (see Fig. S1 in the supplemental material) in a publicity available data set, Pancan-meQTL (25). This analysis indicated that the haplotype (represented by rs1800796, rs1524107, and rs2066992) prevalent in East Asia was associated with lower levels of *IL-6*, while the Caucasian-common haplotype (represented by rs1800795 and rs1800797) was associated with higher expression of *IL-6*.

10.1128/mBio.01372-21.1 FIG S1 Promoter methylation of rs18000796 in thymoma. Genotype of rs1800796 (chr7:22766246, hg19) and the methylation levels at probe cg26061582 (chr7:22766209, hg19) in thymoma (THYM). Data are from Pancan-meQTL. Download FIG S1, TIF file, 0.2 MB. Copyright © 2021 Chen et al. 2021 Chen et al. https://creativecommons.org/licenses/by/4.0/ This content is distributed under the terms of the Creative Commons Attribution 4.0 International license.

### Variant alleles for rs1800796, rs1524107, and rs2066992 represent reduced risk of developing severe COVID-19.

Among the three promoter SNPs that correlate with *IL-6* expression, rs1800797 and rs1800795 are exclusively ancestral *G*-*G* genotype in East Asians. Another promoter SNP, rs1800796, and its strongly linked intronic SNPs, rs1524107 and rs2066992, were highly polymorphic in Asians (Fig. 1c). Therefore, we assessed the genetic variations of the three loci in Chinese COVID-19 patients with mild or severe illness. A total of 105 COVID-19 cases from areas where it was not endemic (Guangdong and Guizhou province), without treatment delay, and 149 healthy controls matched for matched for age, sex, and geographic origin were enrolled in this study. Their characteristics are shown in Table 1 and Table S1. Similar to other reports, age, sex, and underlying health conditions, such as diabetes, hypertension, and coronary artery disease, were risk factors for severe COVID-19. The median age of severe COVID-19 was 18 years older than that for the patients with mild illness (58 versus 40 years old). Men were at greater risk of more severe COVID-19 outcomes than women, with the male-to-female ratio being 3:2 in severe cases and 1:1 in mild cases (Table 1 and Table S1). The factors underlying men’s extra vulnerability might be due to the sex differences in the immune response. For example, males are known to produce more IL-6 than females partly because sex hormones impact IL-6 expression (26, 27).

**TABLE 1:** Age, sex, and demographic characteristics of COVID-19 patients with severe and mild symptoms

Group	Value for:		*P* value^*a*^
	Severe cases (*n* = 35)	Mild cases (*n* = 70)	
Age, yr, median (range)	58 (26–84)	40 (23–77)	<0.001
Sex, no. (%)			0.333
Male	21 (60.0)	35 (50.0)	
Female	14 (40.0)	35 (50.0)	
Hypertension, no. (%)	15 (42.9)	3 (4.3)	<0.001
Coronary artery disease, no. (%)	5 (14.3)	0 (0)	0.003
Diabetes, no. (%)	8 (22.9)	2 (2.9)	0.002

a *P* values comparing severe and mild cases are derived from Mann-Whitney U test, Pearson chi-square (χ^2^) test, or Fisher’ exact test.

10.1128/mBio.01372-21.4 TABLE S1 Age, sex, demographic characteristics of COVID-19 cases and healthy controls. *P* values comparing COVID-19 cases and healthy controls are derived from Mann-Whitney U test, Pearson chi-square (χ^2^) test, or Fisher’ exact test. Download Table S1, DOCX file, 0.01 MB. Copyright © 2021 Chen et al. 2021 Chen et al. https://creativecommons.org/licenses/by/4.0/ This content is distributed under the terms of the Creative Commons Attribution 4.0 International license.

We then genotyped rs1800796, rs1524107, and rs2066992 in patients and the healthy matched controls. All genotypes were in Hardy-Weinberg equilibrium. Because of the low frequency of wild-type alleles in East Asians, samples harboring ancestral alleles (rs1800796 *G*/*G* and *G*/*C*, rs1524107 *C*/*C* and *C*/*T*) were combined for analysis in the dominant model. The allele and genotype frequencies were similar between healthy controls and COVID-19 patients (Table S2). However, the mild and severe COVID-19 groups showed different patterns of *IL-6* polymorphisms. The wild-type allele *G* of rs1800796 exhibited a higher frequency in severe cases than mild cases (38.6% versus 22.9%, *P *= 0.017), and because of the high LD of rs1800796 with rs1524107 and rs2066992, the wild-type allele *C* of rs15240107 was also overrepresented in severe cases (37.1% versus 22.9%, *P *= 0.029) (Table 2). Consistent with allelic analysis, rs1800796 *C*/*C* (odds ratio [OR] = 0.264, *P *= 0.009) and rs1524107 *T*/*T* (OR = 0.319, *P *= 0.013) were identified as protective genotypes after adjustment for sex and age (Table 3). At the haplotypic level, 29.0% of severe cases and 58.0% of mild cases were homozygous for the *C-T-T* haplotype (OR = 0.256, 95% confidence interval [CI] = 0.088 to 0.739, *P *= 0.007) (Table 4). These data suggest that the variant *C-T-T* haplotype of rs1800796, rs1524107, and rs2066992 has a protective role with regard to COVID-19 outcome.

**TABLE 2:** Allele frequencies of two *IL6* polymorphisms among COVID-19 patients with severe and mild symptoms^*a*^

Group and allele	No. (%) of cases		χ^2^ value	*P* value
	Severe (*n* = 35)	Mild (*n* = 70)		
rs1800796				
*G*	27 (38.6)	32 (22.9)	5.704	0.017
*C*	43 (61.4)	108 (77.1)		
rs1524107^a^				
*C*	26 (37.1)	32 (22.9)	4.764	0.029
*T*	44 (62.9)	108 (77.1)		

a The statistical analysis was carried out using the Pearson chi-square (*χ*^2^) test.

**TABLE 3:** Genotype frequencies of two *IL6* polymorphisms among COVID-19 patients with severe and mild symptoms^*a*^

Group and genotype	No. (% frequency) of cases		χ^2^ value	*P* value	OR (95% CI)
	Severe (*n* = 35)	Mild (*n* = 70)			
rs1800796					
*G*/*G+G*/*C*	24 (68.6)	29 (41.4)	6.877	0.009	1.000 (reference)
*C*/*C*	11 (31.4)	41 (58.6)			0.264 (0.095–0.732)
rs1524107^*b*^					
*C*/*C+C*/*T*	24 (68.6)	30 (42.9)	6.176	0.013	1.000 (reference)
*T*/*T*	11 (31.4)	40 (57.1)			0.319 (0.118–0.862)

a Data are presented as frequency (percent). *P* values were determined using χ^2^ test. OR and 95% CI were calculated using logistic regression, adjusted for sex and age.

b rs1524107 and rs2066992 are in strong LD with the same allelic frequencies.

**TABLE 4:** Association between COVID-19 severity and major haplotypes of rs1800796, rs1524107, and rs2066992^*a*^

Genotype^a^	No. (%) of cases		χ^2^ value	*P* value	OR (95% CI)
	Severe (*n* = 31)	Mild (*n* = 69)			
Wild-type G-C-G homozygote and heterozygote	22 (71.0)	29 (42.0)	7.168	0.007	1.000 (reference)
Variant C-T-T homozygote	9 (29.0)	40 (58.0)			0.256 (0.088–0.739)

a Data are presented as frequency (percent). *P* values were determined using χ^2^ test. OR and 95% CI were calculated using logistic regression, adjusted for sex and age. Two rare haplotypes (2 *G*-*T*-*T* and 3 *C*-*C*-*G* alleles) were not included due to low frequencies.

10.1128/mBio.01372-21.5 TABLE S2 Comparison of the allele and genotype frequencies of *IL-6* SNPs between COVID-19 patients and healthy controls (HC). The statistical analysis was carried out using the Pearson chi-square (*χ*^2^) test. rs1524107 and rs2066992 are in strong linkage disequilibrium (LD) with the same allelic frequencies. Download Table S2, DOCX file, 0.01 MB. Copyright © 2021 Chen et al. 2021 Chen et al. https://creativecommons.org/licenses/by/4.0/ This content is distributed under the terms of the Creative Commons Attribution 4.0 International license.

### Genotype of the susceptibility loci determines CTCF binding at the *IL-6/IL-6-AS1* locus.

To determine whether the risk-associated loci we identified were functionally relevant, we sought to characterize genotype-specific changes in protein binding to the risk loci. The candidate SNP rs1800796 is located in the promoter of *IL-6*, and rs1524107 and rs2066992 are located in intron 2 of *IL-6* and the transcription regulatory region of *IL-6-AS1*, a long noncoding RNA (lncRNA) that overlaps *IL-6* on the antisense strand. The other two candidate SNPs linked with rs1800796, rs1524107, and rs2066992 are intronic in *IL-6* and upstream of *IL-6-AS1* (Fig. 2a). The three susceptibility SNPs did not locate in any known transcription factor binding sites according to previous studies (19) (Fig. 1b) and prediction approaches. We then characterized the genotype-specific epigenetic changes around the risk loci. By analyzing the regulatory potential of these loci using ENCODE data in the UCSC Genome Browser (28), we found the three candidate loci reside at CCCTC-binding factor (CTCF) binding regions (Fig. 2b). CTCF is an architectural protein that helps establish the three-dimensional organization of the eukaryotic genome and regulates gene expression in various ways (29), and a recent report suggests that CTCF is required for full-blown upregulation of inflammatory genes, including *IL-6*, in acute inflammatory response (30). Analysis of publicly available chromatin immunoprecipitation sequencing (ChIP-seq) data in multiple cell lines revealed that CTCF binding to two regions in the *IL-6*/*IL-6-AS1* locus encompassed rs1800796, rs1524107, and rs2066992 (Fig. 2b). Scanning of CTCF binding motifs by Find Individual Motif Occurrences (FIMO) found two CTCF binding sites within the human *IL-6*/*IL-6-AS1* locus. Binding site 1 was located upstream of the promoter SNPs rs1800797 and rs1800796, but the sequences of this binding site were not conserved in mammals (Fig. 2c, top). Binding site 2, located in the intron region encompassing rs2066992, and the sequences of this motif were highly evolutionarily conserved, indicating the second CTCF-binding motif has more conserved functional roles across mammals than the first binding motif (Fig. 2c, bottom). Consistent with publicly available ChIP-seq data sets, the binding of CTCF to the two target regions was verified in our ChIP assay in peripheral blood mononuclear cells (PBMCs) from healthy donors (Fig. 2d).

Notably, rs2066992 was located in the second CTCF binding motif of *IL-6*. Wild-type allele of rs2066992 is a cytosine (C) residue at position 12 of the CTCF consensus sequence on the reverse strand orientation of *IL-6* (forward strand orientation of *IL-6-AS1*). *C* to *A* variation was predicted to disrupt CTCF binding to that motif (Fig. 2c, bottom). Indeed, the presence of the second CTCF ChIP-seq peak in the *IL-6*/*IL-6-AS1* locus seemed to be polymorphism dependent. Cell lines derived from Africans and Caucasians with wild-type genotypes at the three SNPs showed two evident CTCF binding peaks, such as HeLa-S3 (African), MCF7 (Caucasian), A549 (Caucasian), and K562 (Caucasian) (Fig. 2b, top). In sharp contrast, the CTCF binding peaks, especially the second peak encompassing rs2066992, were largely diminished in cell lines derived from East Asians with a homozygous *IL-6* variant allele genotype, such as Ishikawa and KU812 (Fig. 2b, bottom). The heterozygote behaved as the wild-type homozygote, such as Kasumi-1, which was also derived from Asians (Fig. 2b, middle). The genotype of each cell line is listed in Table S3.

10.1128/mBio.01372-21.6 TABLE S3 Genotype of the IL-6 SNPs using the CTCF ChIP-seq data of the cell lines. NA, not available. Susceptibility loci identified in this study are in boldface. Download Table S3, DOCX file, 0.01 MB. Copyright © 2021 Chen et al. 2021 Chen et al. https://creativecommons.org/licenses/by/4.0/ This content is distributed under the terms of the Creative Commons Attribution 4.0 International license.

We also generated ChIP-seq data from LCLs to ascertain the CTCF binding disparities were indeed genotype but not cell type dependent. Consistent with this, LCLs established from Chinese donors with variant genotype (Donor1 and Donor2) lost the second CTCF binding peak, which was intact in GM12878, an LCL derived from a Caucasian with wild-type genotype at the candidate susceptibility loci (Fig. 2e). Next, we verified the genotype-dependent CTCF binding in healthy donors grouped by genotypes of the 3 loci. In line with the ChIP-seq data, the binding of CTCF to the *IL-6* intron region (target 2) was diminished in PBMCs of individuals who were homozygous for the variant haplotype *C*-*T*-*T* (Fig. 2f, right). In contrast, the first CTCF binding peak within the *IL-6* promoter region (target 1) was not obviously affected by the genotypes of these *IL-6* SNPs (Fig. 2f, left), likely because none of the SNPs were located in or in close proximity to the first CTCF binding motif of *IL-6* (Fig. 2c). *In vivo*, carriers with the homozygous *C*-*T*-*T* genotype were found to have the lowest *IL-6-AS1* expression, followed by the heterozygous genotype, and the wild-type *G*-*C*-*G* homozygote had the highest expression (Fig. 2g). These results suggest that the risk-associated SNPs prevalent in Asian populations regulate *IL-6/IL-6-AS1* expression by controlling CTCF-mediated chromatin interactions.

### The protective variant haplotype associated with reduced transcription of *IL-6*’s antisense transcript *IL-6-AS1*.

**FIG 3:**
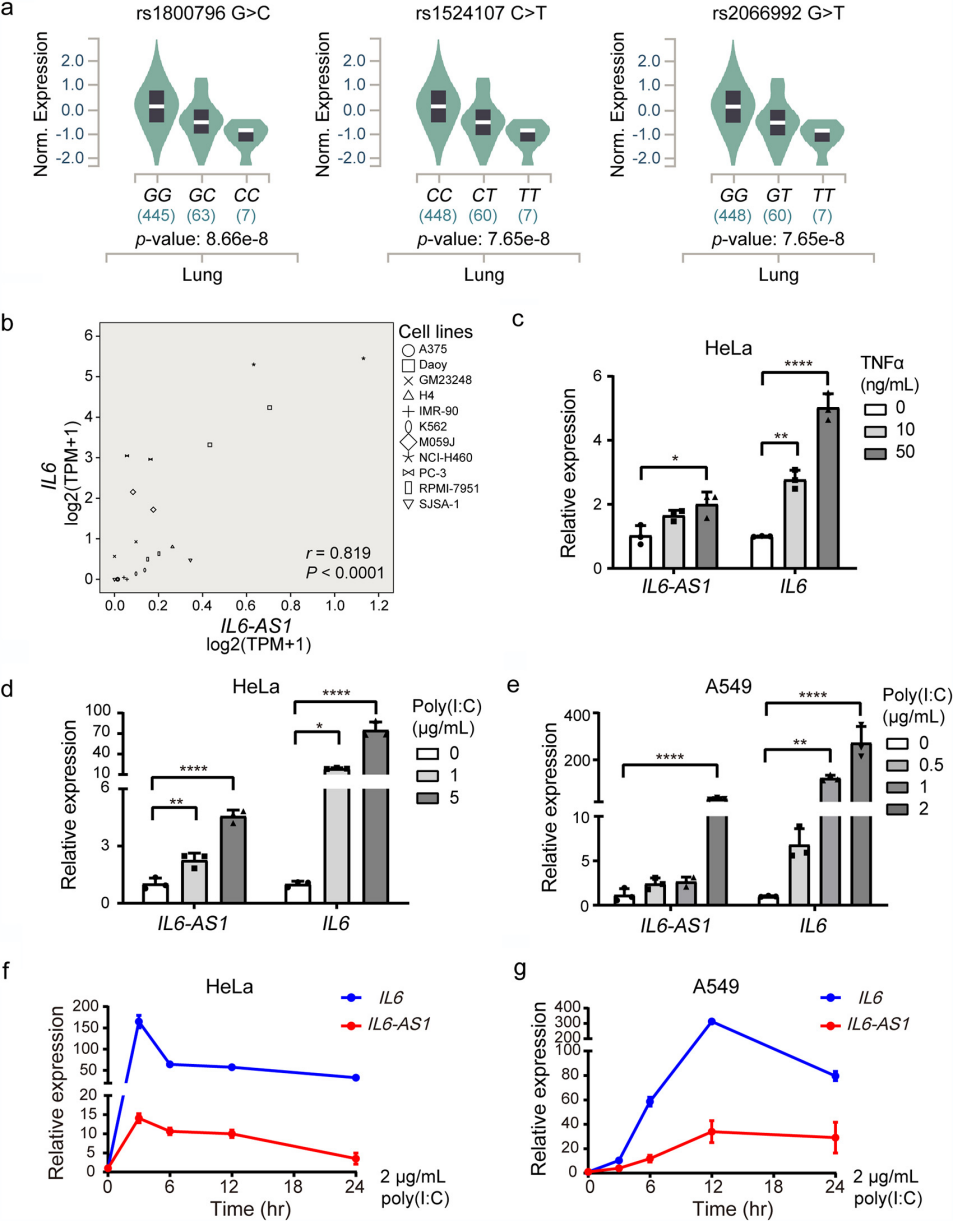
Association between *IL-6* polymorphisms and the expression of *IL-6* and its antisense transcript lncRNA *IL-6-AS1*. (a) Expression quantitative trait locus (eQTL) analysis of association trends for rs1800796, rs1524107, rs2066992, and *IL-6-AS1* expression in lung tissues. All data were collected from the GTEx project. (b) Pearson correlation analysis between *IL-6-AS1* and *IL-6* mRNA expression in several cell lines. *r*, Pearson correlation coefficient. (c to e) mRNA expression of *IL-6-AS1* and *IL-6* in HeLa (c and d) and A549 (e) cells. Cells were treated with TNF-α (c) or transfected with poly(I·C) (d and e) for 12 h. One-way ANOVA with Bonferroni correction for multiple comparisons was used. *, *P *< 0.05; **, *P *< 0.01; ***, *P *< 0.001. (f and g) Time course expression of *IL-6* and *IL-6-AS1* following poly(I·C) transfection into HeLa and A549 cell line. Data represent means ± SD per group.

Given that the rs2066992-containing CTCF binding motif is located upstream of the *IL-6-AS1* coding region but in the intron of *IL-6* (Fig. 2a), we speculated that the rs2066992 *C* to *A* variation would primarily affect *IL-6-AS1* expression. We analyzed the Genotype-Tissue Expression (GTEx) status of the three SNPs (rs1800796, rs1524107, and rs2066992) in the lung tissue, and the *cis*-expression quantitative trait loci (eQTL) of three SNPs were associated with expression for *IL-6-AS1* but not for *IL-6* in the GTEx portal, likely because *IL-6* level is highly unstable in postmortem tissues. Using transcriptome sequencing (RNA-seq) data of 515 individuals worldwide from the GTEx project (31), we found the genotypes of the three SNPs strongly associated with *IL-6-AS1* expression. The protective genotype carriers were associated with lower expression of *IL-6-AS1* in an allelic dose-dependent manner (i.e., the lowest for the variant homozygote) (Fig. 3a). Because the variant allele of rs1800796 also associated with lower expression of *IL-6* (15, 32) (Fig. 1f), we hypothesized that the three SNPs would affect the transcription of both *IL-6* and *IL-6-AS1* simultaneously. Indeed, a strong correlation between *IL-6-AS1* and *IL-6* levels was observed in several cell lines via analysis of their transcriptomic data from the ENCODE project (*r* = 0.819, *P *< 0.0001; Fig. 3b). We also examined the profiles of *IL-6-AS1* and *IL-6* expression with inflammatory stimulators, including tumor necrosis factor alpha (TNF-α) and poly(I·C), an analogue of exogenous RNA that mimics RNA virus infection. The results suggested that TNF-α and poly(I·C) upregulated *IL-6-AS1* and *IL-6* in a dose-dependent manner (Fig. 3c to e). Measurement of the time course also showed rapid poly(I·C) induction in *IL-6-AS1* and *IL-6* expression that peaked at ∼3 h in HeLa cells (Fig. 3f) and ∼12 h in A549 cells (Fig. 3g). Thus, the induction of the lncRNA *IL-6-AS1* mirrored that of the nearby gene *IL-6*, although the upregulation of *IL-6-AS1* was less robust than *IL-6* in inflammation response.

### The protective variant haplotype impairs *IL-6* induction upon inflammatory stimuli.

**FIG 4:**
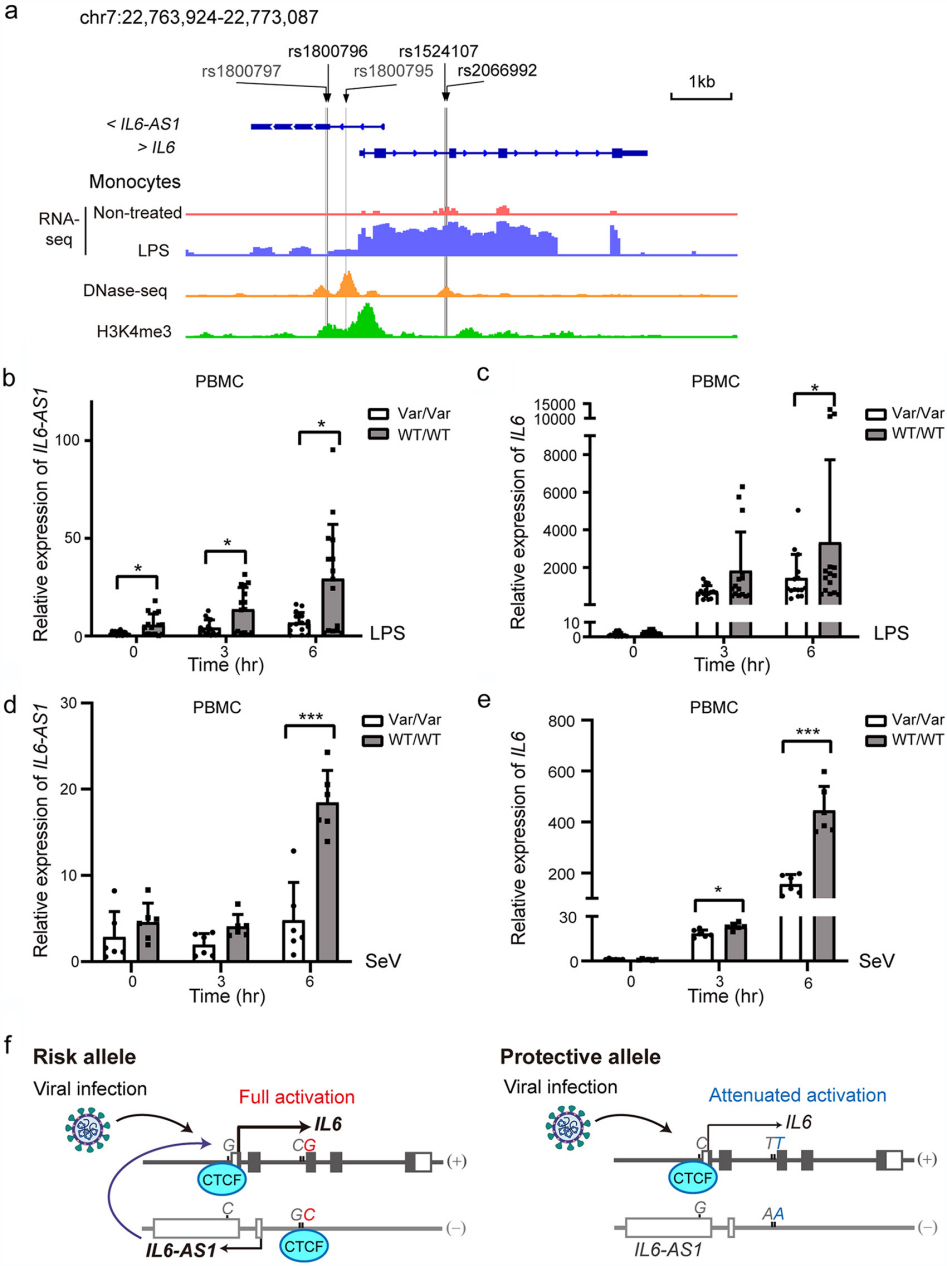
Characterization of SNP-regulated *IL-6/IL-6-AS1* expression upon inflammatory stimuli. (a) Promoter and enhancer signatures at the *IL-6*/*IL-6-AS1* locus. RNA-seq, DNase I, and H3K4me3-seq profiles of the risk-associated SNPs in the *IL-6*/*IL-6-AS1* locus. The position of the 3 promoter SNPs and 2 intronic SNPs are indicated, and the Caucasian-common SNPs are shown in gray. (b and c) mRNA levels of *IL-6-AS1* (b) and *IL-6* (c) following LPS treatment at the indicated times in PBMCs with WT or variant genotypes. Each assay was performed for each donor (WT/WT, *n* = 5; Var/Var, *n* = 5) in technically triplicate. (d and e) mRNA expression of *IL-6-AS1* (e) and *IL-6* (f) upon SeV infection in PBMCs. Each assay was performed for each donor (WT/WT, *n* = 2; Var/Var, *n* = 2) in technical triplicate. Data represent means ± SD per group. Data were analyzed by multiple *t* test with Bonferroni correction for multiple comparisons. *, *P *< 0.05; ***, *P* < 0.001. (d) Genetic polymorphisms of *IL-6* in Asian populations underlie vulnerability to COVID-19. (Left) Viral infections stimulate bidirectional transcription of the *IL-6*/*IL-6-AS1* locus in individuals carrying the wild-type alleles at rs1800796, rs1524107, and rs2066992 loci. Upregulated *IL-6-AS1* acts in *cis* to further enhance the transcription of *IL-6*. High levels of IL-6 exacerbate the excessive inflammatory response and are linked to worse clinical outcome of COVID-19. (Right) The variant allele disrupts CTCF binding at the enhancer element of *IL-6-AS1*, leading to transcriptional inactivation of *IL-6-AS1* and reduced upregulation of *IL-6* in response to an acute SARS-CoV-2 infection. The motif-altering SNP rs2066992 is marked by red (ancestral allele) or blue (derived allele).

Based on their genomic position and the strong correlation with *IL-6-AS1* expression, variations at the risk-associated loci, particularly rs1524107 and rs2066992, are highly likely to affect the promoter or enhancer activity of the lncRNA *IL-6-AS1*. To distinguish whether the putative lncRNA loci rs1524107 and rs2066992 functioned as enhancers of DNA elements or as lncRNA promoters, H3K4me3 ChIP-seq and DNase-seq data in CD14^+^ resting monocytes were downloaded from the ENCODE database, and the read coverage surrounding the candidate SNPs was assessed. The H3K4me3 signature in the promoter region often defines the promoter, and enhancer elements are marked by DNase hypersensitivity or H3K27Ac but do not overlap a promoter. Using these criteria, rs1524107 and rs2066992 were considered enhancer elements because the two loci are marked by a DNase hypersensitivity signature (Fig. 4a, yellow) but lack H3K4me3 signal (Fig. 4a, green). In contrast, the three promoter SNPs (rs1800795, rs1800796, and rs1800797) did locate at the active promoter, as H3K4me3 signals were enriched at these loci. Of note, the DNase I signal exhibited positional overlap with the CTCF-binding peaks, indicating that CTCF binding at the *IL-6*/*IL-6-AS1* locus functions as an enhancer of *IL-6-AS1* rather than an insulator or repressor in this context (29). The epigenetic data suggest that variations at the lncRNA locus rs2066992, and likely rs1524107 locus, disrupt the CTCF-mediated enhancer-promoter interactions and the establishment of the functional domain of *IL-6-AS1*.

We noticed the differences in baseline serum IL-6 levels were not significant among individuals with different genotypes at the three loci (Fig. S2 and Table S4). In fact, like other inflammatory genes, *IL-6* expression was very low under unstimulated conditions but robustly upregulated by inflammatory stimuli and viral infections. It has been shown that *IL-6* is significantly affected by its neighboring lncRNA *IL-6-AS1* under lipopolysaccharide (LPS) treatment (33). We tried to determine whether the genotypes of the candidate loci affect the transcriptional activation of *IL-6* under stimulated conditions. To this end, PBMCs of healthy donors were treated with LPS for different times, and the expression of *IL-6* and *IL-6-AS1* pair was determined by quantitative PCR (qPCR). We found that the induction of *IL-6-AS1* was most significantly affected by the genotypes of the donor, and the induction extent in the wild-type carriers was over 3-fold higher than the carriers with homozygous variant genotype (Fig. 4b). On the other hand, the upregulation of *IL-6* upon LPS stimulation was also affected by the candidate polymorphisms, and the induction extent in the WT genotype was over 2-fold higher than the variant counterpart (Fig. 4c). In addition, we investigated the effect of *IL-6* genotype on *IL-6*/*IL-6-AS1* expression after RNA virus infection. As primary PBMCs are notoriously hard to transfect, we were unable to detect the effect of the RNA virus mimic poly(I·C) in PBMCs. Alternatively, we chose to infect with Sendai virus (SeV), an RNA virus, to stimulate *IL-6* synthesis in PBMCs. Consistent with these findings, the extent of *IL-6-AS1*/*IL-6* induction was higher in the variant donors than in the WT donors (Fig. 4d and e). Therefore, the haplotype comprised of rs1800796, rs1524107, and rs2066992 influences *IL-6* expression via affecting the transcription of its antisense RNA *IL-6-AS1*, especially in acute inflammation and viral infection.

10.1128/mBio.01372-21.2 FIG S2 Comparison of plasma IL-6 levels at baseline of healthy donors with different genotypes at rs1800796, rs1524107, and rs20669992 loci. The plasma concentrations of IL-6 were determined using IL-6 ELISA kit (ABclonal number RK00004) according to the manufacturer’s protocols. Donors in each group were matched for sex and age as listed in Table S2. Data represent the means ± SD per group. Data were analyzed by Kruskal-Wallis test, and IL-6 expression was not statistically significant for each group. Download FIG S2, TIF file, 0.2 MB. Copyright © 2021 Chen et al. 2021 Chen et al. https://creativecommons.org/licenses/by/4.0/ This content is distributed under the terms of the Creative Commons Attribution 4.0 International license.

10.1128/mBio.01372-21.7 TABLE S4 Characteristics of healthy donors for plasma IL-6 testing. Download Table S4, DOCX file, 0.01 MB. Copyright © 2021 Chen et al. 2021 Chen et al. https://creativecommons.org/licenses/by/4.0/ This content is distributed under the terms of the Creative Commons Attribution 4.0 International license.

Based on the above-described evidence, we concluded that in WT carriers, viral infection or tissue damage-induced inflammatory response triggered bidirectional activation of the *IL-6/IL-6-AS1* locus, resulting in full activation of *IL-6* through direct promoter activation and the lncRNA-mediated transcription of closely located genes in *cis* (Fig. 4f, left); in individuals carrying the variant allele that disrupting CTCF-binding locus at the enhancer of *IL-6-AS1*, the impact of *IL-6-AS1* on *IL-6* upregulation was minimal, and *IL-6* is mainly regulated by its promoter activation. As a result, *IL-6* was only modestly elevated upon inflammatory stimulation (Fig. 4f, right).

### Evolutionary origin of the risk-associated rs1800796, rs1524107, and rs2066992 polymorphisms.

Given IL-6 plays a general role in host defense against pathogens, the evolutionary origins of rs1800796, rs1524107, and rs2066992 variations, which are carried in greater than 76% of modern East Asians and at high frequencies in other populations of Asian descent, are worthy of investigation. Comparing the sequences in mammalian species suggested that except rs1524107, the sequences of the other 4 loci are highly conserved among mammals, and all species carried ancestral sequences (Table 5), indicating the SNPs of interest are human lineage specific.

**TABLE 5:** Conservation of the *IL6* SNPs associated with gene expression in mammalian species

Species	SNP ID				
	rs1800797	rs1800796	rs1800795	rs1524107	rs2066992
Homo sapiens	G/A	G/C	G/C	C/T	G/T
Chimp	G	G	G	C	G
Rhesus	G	G	G	C	G
Cow	G	G	G	A	G
Sheep	G	G	G	A	G
Dog	G	G	G	A	G
Rat	G	G	G	G	G
Mouse	G	G	G	G	G

Interestingly, a recent study reported a Neanderthal-derived region of chromosome 3 as the major genetic risk factor for severe COVID-19 in European and South Asian populations (4). Archaic humans such as Neanderthals (occupied Europe and Western Asia) and Denisovans (ranged from Siberia to Southeast Asia) contribute genetically to the immune systems of modern human outside Africa (34). This Neanderthal risk haplotype occurs at relatively high frequency in South Asia (30%) and Europe (8%) but is almost absent from East Asia (4), so it is unlikely to contribute COVID-19 susceptibility in East Asian populations. We then investigated whether the risk-associated *IL-6* polymorphisms occurring at high frequency in modern East Asia were inherited from any ancient humans. Genomic DNA data for Neanderthals and Denisovans showed that both hominids harbored ancestral sequences at the 5 SNPs (Table 6). Next, we analyzed the genome data of early humans who inhabited the East Asia mainland. A 40,000-year-old human from Tianyuan Cave outside Beijing, China, yielded the oldest human genome sequence for early East Asians on record. The Tianyuan individual was derived from a population that was ancestral to many present-day Asians and Native Americans (35). Interestingly, the Tianyuan man was heterogeneous at rs1524107 and rs2066992 and carried a *T*-*T* variant allele, suggesting this derived allele occurred 40,000 years ago in East Asia. The Tianyuan man has affinity to Native American populations. Indeed, a 12,000-year-old individual from North America (Anzick–1) was found to be a *C*-*T*-*T* homozygote. GoyetQ116–1, a 35,000-year-old European individual who shares more alleles with the Tianyuan man than other ancient Europeans, carried the variant *T*-*T* allele at rs1524107 and rs2066992 (Table 6). GoyetQ116–1 did not contribute to ancestry of present-day Europeans (35), in agreement with the low frequency of this variant haplotype in modern Europeans (Fig. 1c). We also analyzed the recently published genomic data of prehistoric humans who lived 4,000 to ∼8,000 years ago in the China mainland (36). Among 4 individuals with reads at the candidate polymorphic loci, all carried the variant *T*-*T* allele at rs1524107 and rs2066992 (no reads at the promoter SNPs rs1800795, rs1800796, and rs1800797), and 3 out of 4 were likely to be *T*-*T* homozygote (Yumin, LD1, and L5705) (Table 6), supporting the continuity of these *IL-6* polymorphisms in human history in East Asia. The ancient DNA evidence supports the idea that variations at rs1524107 and rs2066992 loci that disrupt an evolutionarily conserved CTCF binding site were derived from archaic humans who were ancestral to present-day mainland Asians and Native Americans. Unlike the Neanderthal risk haplotype (4), the *IL-6* haplotype from ancient East Asians seems to have positive consequences for SARS-CoV-2 infection.

**TABLE 6:** Risk-associated *IL6* polymorphisms in ancient humans

Individual	Accession no.	SNP ID^*a*^					Region	Years ago
		rs1800797	rs1800796	rs1800795	rs1524107	rs2066992		
Modern human		G/A	G/C	G/C	C/T	G/T	Worldwide	Present
Altai Neanderthal	ERP002097	G	G	G	C	G	Russia	>50,000
Denisovan	ERP001519	G	G	G	C	G	Russia	>50,000
Tianyuan	ERR1910473	NA	NA	NA	C_3_/T_1_	G_4_/T_2_	Beijing, China	40,000
Anzick–1	SRX381032	G_24_	C_28_	G_24_	T_12_	T_10_	North America	12,707–12,556
GoyetQ116–1	ERR1341815	NA	NA	G_6_	T_1_	T_1_	Belgium	35,160–34,431
Yumin	HRR051935	NA	NA	G_19_	T_2_	T_2_	Inner Mongolia, China	8,415–8,335
BS	HRR051937	NA	NA	G_33_	C_1_	G_4_/T_2_	Shandong, China	8,320–8,040
LD1	HRR051943	NA	NA	G_5_	T_3_	T_4_	Liang Island, China	8,320–8,060
L5705	HRR051947	NA	NA	G_2_	T_1_	T_3_	Fujian, China	4,419–4,246

a NA, not available. The subscripts refer to the number of reads at each locus.

In summary, this study provides evidence that an Asian-common *IL-6* variant haplotype correlated with the lower production of IL-6 upon inflammatory stimuli, which provides a mechanism for the observed reduced risk of severe COVID-19 illness in patients carrying the variant alleles at rs1800796, rs1524107, and rs2066992 loci. It is conceivable that individuals with the low-producing variant genotype would avoid the prolonged and uncontrolled IL-6 synthesis in the disease progression of COVID-19 (Fig. 4f).

## DISCUSSION

Genetic differences underlie the differential susceptibility to infectious diseases. Numerous studies have shown that *IL-6* polymorphisms were linked to the outcome of viral infections by affecting IL-6 protein production (17, 20, 21, 37, 38). COVID-19 is a pulmonary disease caused by SARS-CoV-2 that exhibits varied severity, ranging from asymptomatic, mild to severe, and life-threatening lower respiratory tract infections, including the development of ARDS (39). Elevated serum concentration of IL-6 is a hallmark of severe COVID-19, and the serum concentration of IL-6 is a predictive biomarker for disease severity of COVID-19 (7, 12–14). These clinical observations suggest that *IL-6* genotype underlies the differential outcomes of SARS-CoV-2 infection. The present study reported a novel association of SNPs rs1800796, rs1524107, and rs2066992 at the *IL-6* locus with COVID-19 severity in a Chinese population. The wild-type haplotype *G-C-G* predisposed individuals to severe COVID-19, and the variant haplotype *C-T-T* was the protective allele. Variations of the three highly linked SNPs were associated with reduced expression and attenuated the induction of *IL-6* and its antisense lncRNA *IL-6-AS1* by poly(I·C) and LPS treatment, suggesting the variant haplotype plays a protective role against severe COVID-19 by preventing excessive IL-6 production. To our knowledge, this study is the first report on *IL-6* polymorphisms in clinical presentations of COVID-19 in Asian populations.

IL-6 is a pleiotropic cytokine that has anti-inflammatory and proinflammatory functions. Studies using an *IL-6* knockout mouse model suggested that *IL-6* was essential for viral clearance and/or T- and B-cell responses against influenza virus, vaccinia virus, and lymphocyte choriomeningitis virus (37). However, numerous pieces of clinical and *in vivo* evidence suggest negative consequences of the exaggerated synthesis of IL-6 on the immune response. Increased systematic IL-6 was observed in patients infected with Andes virus, influenza virus, HBV, HCV, HIV, and coronaviruses SARS-CoV-2, SARS-CoV, and MERS (8–11, 37). High levels of IL-6 are often associated with the acute severe systemic inflammatory response known as a cytokine storm, and persistent elevation of IL-6 is a predictive factor of poor prognosis of patients with ARDS (40) and COVID-19 (7, 12–14). Because of the conflicting effects of IL-6 during the progression of different infectious diseases, it is not surprising that the protective allele (rs1800796-*C*, rs1524107-*T*, and rs2066992-*T*) for COVID-19 identified in this study was the risk allele for blastomycosis in individuals of Hmong ancestry, in which high levels of IL-6 are beneficial for the development of antifungal T helper 17 (Th17) cells (15). This low-producing *IL-6* genotype was also detrimental for the spontaneous clearance of HBV and increased the risk of chronic HBV infection (16, 17, 41), largely due to the inhibitory role of IL-6 in HBV entry and replication (38). The prevalence of this variant genotype in East Asia may partially explain why China has the world’s largest burden of HBV infection (42). Considering the known plethoric effects of IL-6 on immunological outcomes, we expect polymorphisms at these loci to influence other infectious diseases.

The susceptibility loci we identified regulate *IL-6* expression mainly by affecting the regulatory antisense lncRNA *IL-6-AS1*. The intronic SNP rs2066992-*T* allele disrupted a very conserved CTCF binding site at the enhancer elements of *IL-6-AS1* (Fig. 2), an antisense transcript of *IL-6* that mirrors the expression of *IL-6* (15, 32) (Fig. 3). lncRNAs including *IL-6-AS1* have been shown to influence the expression of a neighboring protein-coding gene in *cis* during the inflammatory response (43, 44). Furthermore, a recent study reported that CTCF-dependent genome organization is required for rapid and robust activation of inflammatory genes, including *IL-6*, in an acute inflammatory response (30). In this study, we found that carriers with the variant genotype were associated with lower expression of *IL-6-AS1* under both unstimulated and stimulated conditions, while the IL-6 level was only affected by the genotype after inflammatory stimuli (Fig. 4). Given the pivotal role of CTCF in the formation and maintenance of three-dimensional (3D) chromatin structure, the CTCF motif-altering SNP may fine-tune *IL-6* expression mainly via differentially organized chromatin interactions in inflammatory conditions (30). Chromatin interaction analysis by paired-end tag sequencing (ChIA-PET) assay is an emerging technology to study long-range chromatin interactions (45). Using CTCF ChIA-PET data from the GM12878 cell line, we found that the *IL-6* locus had strong remote interactions with multiple genes, including STEAP1B, AC002480.1, AC002480.2 (lncRNAs antisense to STEAP1B), and a pseudogene, AC073072.2 (see Fig. S3 in the supplemental material). Due to a lack of chromatin interaction data derived from individuals carrying the variant genotype at the three loci, the exact chromatin loop(s) and promoter-enhancer interactions disrupted by the motif-altering SNP are currently unknown. Future epigenetic studies, including spatial contact maps derived from different genetic backgrounds before and after inflammatory stimulations, will be needed to fully understand the role of the Asian-common haplotype on the *IL-6* system.

10.1128/mBio.01372-21.3 FIG S3 Snapshots depicting the interconnectivity of CTCF chromatin interactions at IL-6 and its surrounding genes in GM12878. From top to bottom, locations of three SNPs (rs1800796, rs1524107, and rs2066992), gene annotation track from 22580 kb to 22830 kb of chr7 (human reference genome hg19), CTCF binding peak profile (defined by ChIP-seq, orange track), CTCF interligation peak profile (defined by ChIA-PET, blue track), and CTCF chromatin looping track (defined by ChIA-PET, red track). Accession numbers are ENCFF312KXX (ChIP-seq, ENCODE) and SRR2312566 (ChIA-PET, GEO). Download FIG S3, TIF file, 0.8 MB. Copyright © 2021 Chen et al. 2021 Chen et al. https://creativecommons.org/licenses/by/4.0/ This content is distributed under the terms of the Creative Commons Attribution 4.0 International license.

A major limitation of the present study is the small sample size (*n* = 105), which decreased the statistical power of this study. Further study with a larger sample size must be conducted to obtain conclusive evidence of the association between *IL-6* polymorphisms and IL-6 production and disease severity in Asian COVID-19 patients.

On the other side, GWAS had been carried out in European populations but did not find an association of *IL-6* polymorphisms with severe COVID-19 (4, 5). Instead, a Neanderthal-derived region of chromosome 3 had been suggested to be the major genetic risk factor (4). This haplotype in relatively common in European and South Asian populations but was almost absent from East Asia, indicating this Neanderthal haplotype underwent negative selection in East Asia in the past. *IL-6* polymorphisms vary significantly between Caucasians and Asians. In East Asia, the low-producing *IL-6* haplotype inherited from ancient East Asians, such as Tianyuan man, has beneficial effects with respect to the COVID-19 pandemic (Tables 4 and 6). The exact driving force for this low-producing *IL-6* variant is currently unknown but likely is a pathogen-driven balancing selection to the historical pathogen prevalence in East Asia. In Europe, a high-producing *IL-6* haplotype consisting of rs1800797 (−597 *G* > *A*) and rs1800795 (−174 *G* > *C*) occurs at a frequency of 30 to ∼40% (Fig. 1). rs1800797 and rs1800795 are located in the active promoter marked by H3K4me3 signature (Fig. 4a), and the variant alleles correlated with higher expression of *IL-6* (Fig. 1) (19, 46). Therefore, the haplotype endemic to Europe likely influences *IL-6* expression by simple promoter activation and may be associated with higher expression of *IL-6* in both unstimulated and stimulated conditions, so its regulatory mechanisms are different from those of the haplotype prevalent in East Asia. The net effect of this high-producing Caucasian *IL-6* genotype on COVID-19 outcome might be neutral, as higher basal levels of IL-6 are beneficial for the activation of immune response and viral clearance, but continual synthesis of IL-6 exerts a pathological effect (37), which may explain the lack of *IL-6* polymorphisms association with COVID-19 in European populations.

The SNPs we identified may serve as a useful genetic tool to screen high-risk COVID-19 patients in Asian populations. Because of the high LD, rs1800796 or rs2066992 may be selected as a tag SNP to evaluate the risk of progression to severe symptoms. More attention should be paid to the IL-6 serum levels in patients harboring the high-producing *IL-6* genotype, and appropriate treatment is needed, such as dexamethasone, a glucocorticoid compound that suppresses *IL-6* transcription by binding to GRE at the *IL-6* promoter (47, 48) (Fig. 1b), or a humanized anti-IL-6 receptor antibody that directly inhibits IL-6 signaling, such as tocilizumab. Clinical trials showed that dexamethasone and tocilizumab improved hospital survival of severely ill COVID-19 patients (48–50). We hypothesized that COVID-19 patients carrying the high-producing *IL-6* genotype were more likely to benefit from dexamethasone and IL-6 blockade therapy. In contrast, patients carrying the low-producing genotype should avoid such therapy in the early phase of infection, as it may lead to inefficient immune activation. Therefore, *IL-6* genotyping of rs1800796 could be included in dexamethasone and tocilizumab trials to maximize drug efficacy and safety in Asian populations.

## MATERIALS AND METHODS

### Patient enrollment and sample collection.

The study included 105 laboratory-confirmed COVID-19 cases and 149 healthy controls of Chinese ethnicity who were matched for age, sex, and geographic origin (Guangdong and Guizhou provinces). Their main demographic characteristics are listed in Table 1. The laboratory-confirmed COVID-19 patients (aged ≥22 years) were hospitalized in the First Affiliate Hospital of Guangzhou Medical University (*n* = 25), People’s Hospital of Yangjiang (*n* = 13), Qingyuan People’s Hospital (*n* = 9), and Guizhou Provincial People’s Hospital (*n* = 58) between 26 January and 4 May 2020. Patients with severe pneumonia who were admitted to the intensive care unit and required mechanical ventilation were enrolled in the severe illness group. Patients with a mild clinical presentation (primarily fever, cough, malaise, and headache, including nonpneumonia or mild pneumonia) were enrolled in the mild illness group. Among 105 patients, 35 patients were classified as severe cases, and 70 patients were mild cases. Informed consent was obtained from patients and healthy donors. The present study had IRB approval from the Health Commission of Guangdong Province and the ethics committees of each of the hospitals used to obtain patient and healthy donor samples. Written informed consent was obtained from all participants.

### DNA extraction and SNP genotyping.

Genomic DNA was extracted from blood samples using the QIAamp DNA blood minikit (Qiagen, Germany) according to the manufacturer’s protocol. DNA fragments encompassing the SNPs of interest were amplified from genomic DNA samples, and the amplified PCR products were genotyped using Sanger sequencing.

### Cell culture.

The lung cancer cell line A549 and the cervical cancer cell line HeLa were purchased from ATCC (Manassas, VA) and cultured in DMEM (Dulbecco's modified Eagle medium) with 10% fetal calf serum (GIBCO). Epstein-Barr virus-transformed B lymphoblastoid cell lines (LCLs) from healthy donors were established as previously described (51). Human peripheral blood mononuclear cells (PBMCs) were isolated via differential centrifugation using Ficoll-Paque (GE Healthcare, Shanghai, China) from buffy coats of healthy blood donors. PBMCs and LCLs were cultured in RPMI 1640 supplemented with 10% fetal calf serum (GIBCO). Cells were incubated at 37°C in a humidified environment with 5% CO_2_.

### Quantitative real-time PCR.

Cells were treated with TNF-α (300-01A-10; PeproTech) or LPS (L2880; Sigma) or transfected with poly(I·C) (B5551; Apexbio) using Lipofectamine 3000 (L3000015; Thermo Fisher Scientific) for 12 h or infected with Sendai virus (kindly provided by Deyin Guo) for the indicated time points. Total RNA was isolated from cells using TRIzol reagent (Invitrogen, Carlsbad, CA) according to the manufacturer's protocol and reverse transcribed into cDNA using the PrimeScript 1st-strand cDNA synthesis kit (TaKaRa, Tokyo, Japan). Quantitative real-time PCR was carried out using Hieff qPCR SYBR green master mix (Yeasen, Shanghai, China) with gene-specific primers and cDNA templates according to the manufacturer's protocol. Glyceraldehyde-3-phosphate dehydrogenase (GAPDH) was used as a reference gene. The relative fold change in expression was calculated using the 2^−ΔΔCT^ method. The primer sequences were *IL-6-AS1*-F, CGTCGAGGATGTACCGAATT; *IL-6-AS1*-R, GCAGCACAAGGCAAACCTCT; *IL-6*-F, TCCAGGAGCCCAGCTATGAAC; *IL-6*-R, CAGGACTTTTGTACTCATCTGC; *GAPDH*-F, GATGACCTTGCCCACAGCCT; and *GAPDH*-R, ATCTCTGCCCCCTCTGCTGA.

### ChIP assay.

ChIP in LCLs and human PBMCs were performed as described previously (44). Six to eight million PBMCs were harvested and cross-linked with 1% formaldehyde. Cell lysates were incubated with 1 μg anti-CTCF rabbit antibody (10915-1-AP; Proteintech). Normal rabbit IgG was used as a negative control. Real-time PCR was used to quantify CTCF binding, and data are presented as permillage relative to the input. Primers for qPCR were the following: *IL-6* target 1 primers, −712 bp to −470 bp, *IL-6*-T1-F, CCTCCTCTAAGTGGGCTGAAGC; *IL-6*-T1-R, TGAGTTTCCTCTGACTCCATCG; *IL-6* target 2 primers, +1364 bp to +1535 bp, *IL-6*-T2-F, GGATGCCAATGAGTTGTAGCT; *IL-6*-T2-R, TGCCTCTTTGCTGCTTTCAC; *IL-6* nontarget (NT) primers, +329 bp to +553 bp, *IL-6*-NT-F, ATTCCAAAGATGTAGCCGCCC; *IL-6*-NT-R, CTACAGTGCTCTAGAACCCAGC; and H19 primers, *H19*-F, CCCATCTTGCTGACCTCAC; *H19*-R, AGACCTGGGACGTTTCTGTG. For ChIP-seq analysis, sequenced reads were mapped to the human reference genome hg19 using bowtie2. The signal track visualized on Integrative Genomics Viewer (IGV) was fold enrichment over the control. The signal track of GM12878 (an LCL with homozygous WT genotype at the *IL-6* candidate loci) was downloaded from ENCODE with accession number ENCFF271YKQ. ChIP-seq data of two LCLs harboring the *IL-6* homozygous variant genotype were generated in-house and are available upon request.

### Software and database.

*IL-6* expression data of LCLs was downloaded from the Gene Expression Omnibus (GEO) website with accession number GSE6536. From the 1000 Genomes Project data, we extracted genotypes of 174 individuals whose gene expression data were available in GSE6536. LD analysis was performed on HaploView, and the population frequencies of SNPs were from the 1000 Genomes Project. Visualization of SNP worldwide frequency was done using GGV (http://www.popgen.uchicago.edu/ggv). Processed and normalized data of DNA methylation quantitative trait loci (meQTL) in colorectal cancer (CRC) was acquired from Pancan-meQTL (http://gong_lab.hzau.edu.cn/Pancan-meQTL/). The genotype-specific expression of rs1800796, rs1524107, and rs2066992 in lung tissues via *cis*-expression quantitative trait loci (cis-eQTL) analysis was evaluated using the Genotype-Tissue Expression (GTEx) portal (https://gtexportal.org/home/). *IL-6-AS1* and *IL-6* expression profiles of several cell lines were downloaded from the ENCODE project (https://www.encodeproject.org/). CTCF ChIP-seq data were downloaded from ENCODE and Sequence Read Archive (SRA) (the accession numbers are listed in Table S3 in the supplemental material). Data for H3K4me3 ChIP-seq and DNase-seq in resting CD14^+^ monocytes were downloaded from ENCODE with accession numbers ENCFF231OTU and ENCFF861JZY, respectively. To analyze *IL-6-AS1* and *IL-6* expression profiles, RNA-seq data of human monocytes treated with LPS or nonstimulated (NS) cells were downloaded from study E-MTAB-2399 in the EBI database (https://www.ebi.ac.uk/arrayexpress) with accession numbers ERR458839 and ERR458836, respectively. All sequencing data were visualized using IGV.

Aligned sequences of Altai Neanderthal, Denisovan, Tianyuan, GoyetQ116-1, and Vestonice16 were obtained from the European Nucleotide Archive (ENA; https://www.ebi.ac.uk/ena/browser/home). Sequences of Anzick-1 was acquired from the SRA. Sequences of Yumin, BS, LD1, and L5705 were from the Genome Sequence Archive in the BIG Data Center (GSA; https://bigd.big.ac.cn/gsa-human). The accession numbers for the ancient human genome data are listed in Table 6.

### Statistical analyses.

Pearson correlation analysis was used to evaluate the association between *IL-6-AS1* and *IL-6* expression in several cell lines. The allele and genotype frequencies were determined via direct counting. When comparing baseline information of the subjects, *P* values were determined using Mann-Whitney U test, Pearson chi-square (χ^2^) test, or Fisher’s exact test. The Pearson χ^2^ test was used to compare allele and genotype distributions in patients with mild and severe symptoms. Odds ratios (ORs) and 95% confidence intervals (CIs) were calculated using logistic regression to evaluate the association of *IL-6* genotypes and COVID-19 severity, adjusted for sex and age. All computations were done using SPSS software (version 22.0). Comparisons of *IL-6* expression in different populations and genotypes were performed with nonparametric Kruskal-Wallis tests. Data analysis of CTCF enrichment and *IL-6-AS1* expression in different groups was performed using one-way analysis of variance (ANOVA) with Bonferroni correction for multiple comparisons. Bivariate analyses comparing gene expression in PBMC with different genotypes and LPS stimulation were performed using multiple *t* test with Bonferroni correction for multiple comparisons in GraphPad Prism 8. *P* values of <0.05 were defined as statistically significant. *, *P* < 0.05; **, *P* < 0.01; ***, *P* < 0.001; and ****, *P* < 0.0001.

### Data availability.

The data that support the findings of this study are available upon request.
