# Supplementary figures and images for: A Low-Producing Haplotype of Interleukin-6 Disrupting CTCF Binding Is Protective against Severe COVID-19
Source: mBio. 2021 Oct 12;12(5):e01372-21. doi: 10.1128/mBio.01372-21 (PMC8510538; doi:10.1128/mBio.01372-21)

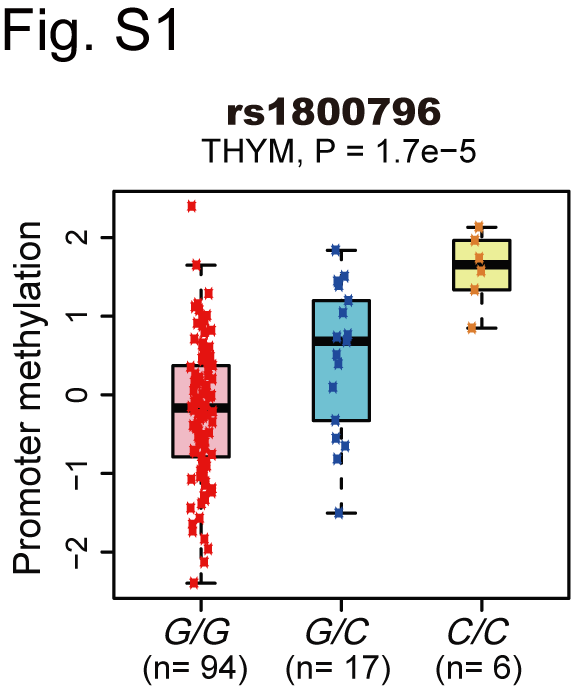

Supplement: FIG S1 [file mbio.01372-21-sf001.tif]

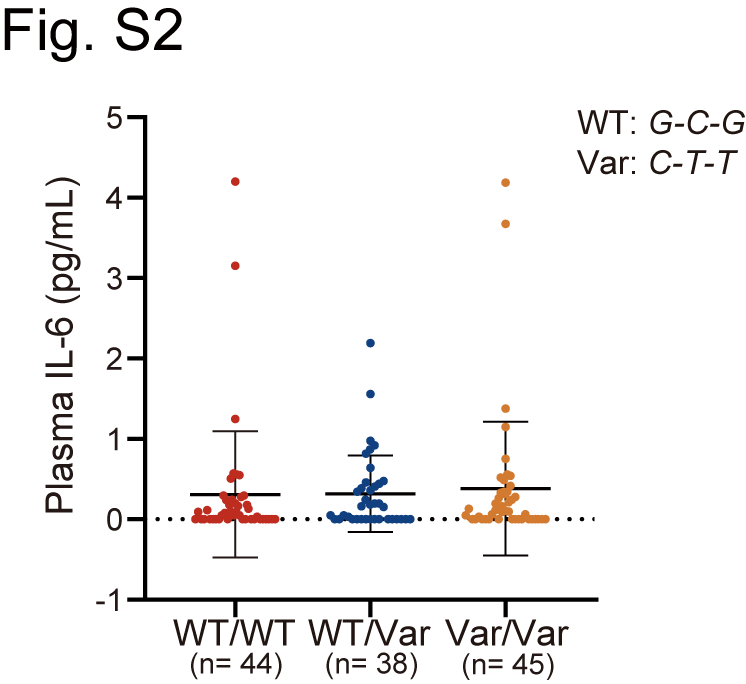

Supplement: FIG S2 [file mbio.01372-21-sf002.tif]

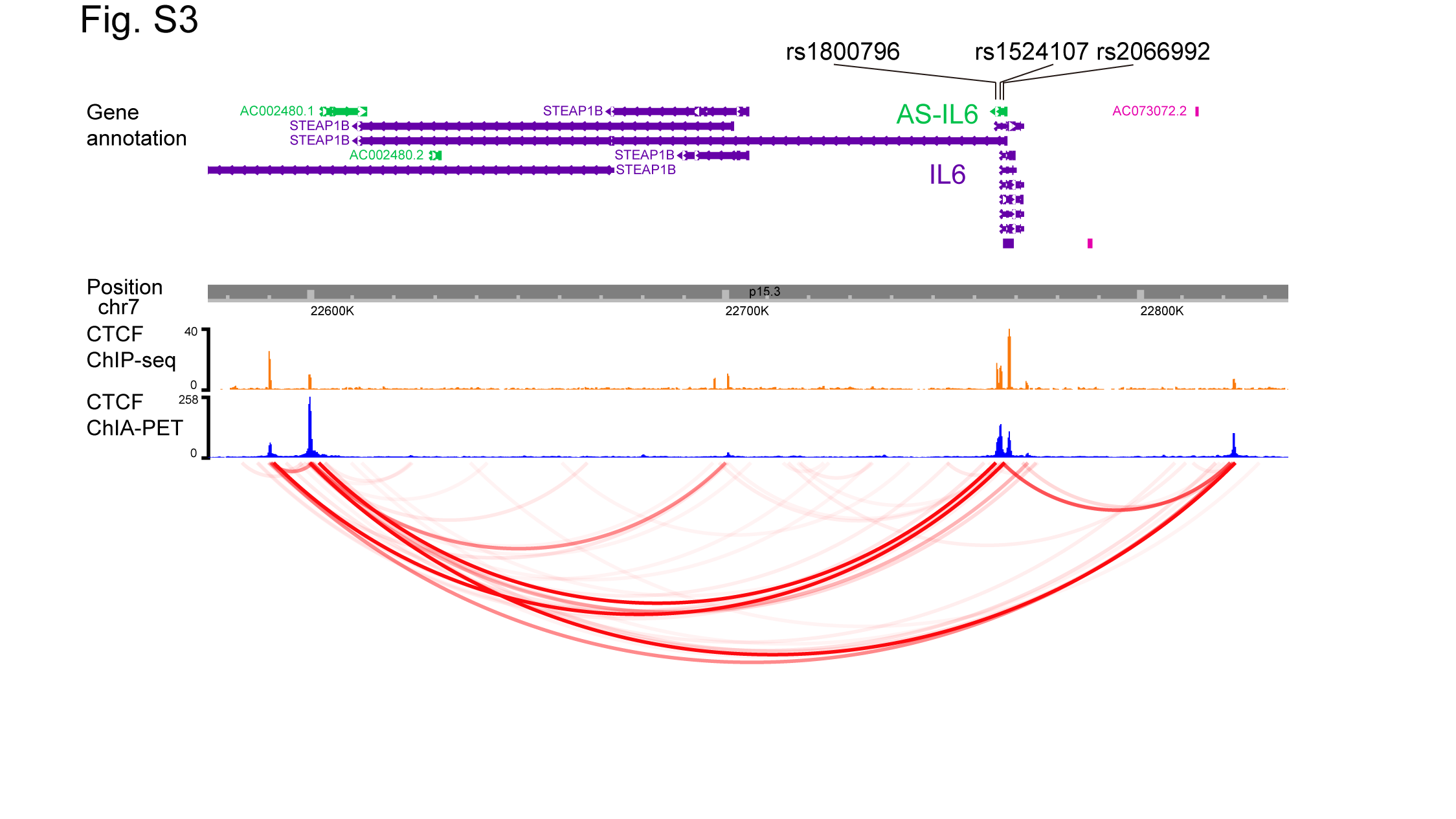

Supplement: FIG S3 [file mbio.01372-21-sf003.tif]
