# Supplementary material for: A Low-Producing Haplotype of Interleukin-6 Disrupting CTCF Binding Is Protective against Severe COVID-19
Source: mBio. 2021 Oct 12;12(5):e01372-21. doi: 10.1128/mBio.01372-21 (PMC8510538; doi:10.1128/mBio.01372-21)
Supplement: TABLE S1 [file mbio.01372-21-st001.docx]

Table S1

| Group | COVID-19 cases  (n = 105) | Healthy controls  (n = 149) | *P* |
| --- | --- | --- | --- |
| Age  Median (range) | 49 (23-84) | 45 (24-78) | 0.071 |
| Sex |  |  | 0.871 |
| Male | 56 (53.3) | 81 (54.4) |  |
| Female | 49 (46.7) | 68 (45.6) |  |
| Hypertension | 18 (17.1) | 3 (2.0) | < 0.001 |
| Coronary artery disease | 5 (4.8) | 0 | 0.011 |
| Diabetes | 10 (9.5) | 1 (0.7) | 0.001 |
