# Supplementary material for: A Low-Producing Haplotype of Interleukin-6 Disrupting CTCF Binding Is Protective against Severe COVID-19
Source: mBio. 2021 Oct 12;12(5):e01372-21. doi: 10.1128/mBio.01372-21 (PMC8510538; doi:10.1128/mBio.01372-21)
Supplement: TABLE S2 [file mbio.01372-21-st002.docx]

Table S2

| Group |  | COVID-19 cases  (n=105) | Healthy controls  (n = 149) | *χ*^2^ | *P* |
| --- | --- | --- | --- | --- | --- |
| rs1800796 |  | Number (% frequency) | |  |  |
| Allele | *G* | 59 (28.1) | 70 (23.5) | 1.379 | 0.240 |
|  | *C* | 151 (71.9) | 228 (76.5) |  |  |
| Genotype | *G*/*G+G*/*C* | 53 (50.5) | 62 (41.6) | 1.954 | 0.162 |
|  | *C*/*C* | 52 (49.5) | 87 (58.4) |  |  |
| rs1524107^a^ |  |  |  |  |  |
| Allele | *C* | 58 (27.6) | 72 (24.2) | 0.774 | 0.379 |
|  | *T* | 152 (72.4) | 226 (75.8) |  |  |
| Genotype | *C*/*C+C*/*T* | 54 (51.4) | 64 (43.0) | 1.779 | 0.182 |
|  | *T*/*T* | 51 (48.6) | 85 (57.0) |  |  |
