# Supplementary material for: A Low-Producing Haplotype of Interleukin-6 Disrupting CTCF Binding Is Protective against Severe COVID-19
Source: mBio. 2021 Oct 12;12(5):e01372-21. doi: 10.1128/mBio.01372-21 (PMC8510538; doi:10.1128/mBio.01372-21)
Supplement: TABLE S3 [file mbio.01372-21-st003.docx]

Table S3

| Cell lines | rs1800797  G>A | **rs1800796**  G>C | rs1800795  G>C | **rs1524107**  C>T | **rs2066992**  G>T | Accession number | Source |
| --- | --- | --- | --- | --- | --- | --- | --- |
| HeLa-S3 | AA | GG | N.A. | CC | GG | ENCFF474UIC | ENCODE |
| MCF7 | AA | GG | N.A. | CC | GG | ENCFF565WPL | ENCODE |
| A549 | AG | GG | N.A. | CC | GG | ENCFF292MAD | ENCODE |
| K562 | AG | GG | N.A. | CC | GG | ENCFF487UYG | ENCODE |
| Kasumi-1 | GG | GC | GG | CT | GT | SRR8060948 | SRA |
| Ishikawa | GG | CC | N.A. | TT | TT | ENCFF100KVY | ENCODE |
| KU812 | GG | CC | N.A. | TT | TT | ERR1068936 | SRA |
