# Supplementary material for: A Low-Producing Haplotype of Interleukin-6 Disrupting CTCF Binding Is Protective against Severe COVID-19
Source: mBio. 2021 Oct 12;12(5):e01372-21. doi: 10.1128/mBio.01372-21 (PMC8510538; doi:10.1128/mBio.01372-21)
Supplement: TABLE S4 [file mbio.01372-21-st004.docx]

Table S4

| Genotype | Sample size (n) | Sex | | Median age (Range) |
| --- | --- | --- | --- | --- |
|  |  | Male (n) | Female (n) |  |
| WT/WT | 44 | 20 | 24 | 32 (19-56) |
| WT/Var | 38 | 17 | 21 | 39 (21-58) |
| Var/Var | 45 | 22 | 23 | 39 (20-59) |
